# The Use of Stem Cells in Burn Wound Healing: A Review

**DOI:** 10.1155/2015/684084

**Published:** 2015-07-07

**Authors:** Fadi Ghieh, Rosalyn Jurjus, Amir Ibrahim, Alice Gerges Geagea, Hisham Daouk, Bassel El Baba, Sana Chams, Michel Matar, Wadih Zein, Abdo Jurjus

**Affiliations:** ^1^Faculty of Medicine, American University of Beirut, Beirut, Lebanon; ^2^Department of Anatomy and Regenerative Biology, George Washington University, Washington, DC, USA; ^3^Department of Surgery, American University of Beirut, Beirut, Lebanon; ^4^Lebanese Health Society, Beirut, Lebanon; ^5^Department of Anatomy, Cell Biology, and Physiology, Faculty of Medicine, American University of Beirut, Beirut, Lebanon; ^6^Eye Institute, National Institutes of Health, Bethesda, MD, USA

## Abstract

Burn wound healing involves a series of complex processes which are subject to intensive investigations to improve the outcomes, in particular, the healing time and the quality of the scar. Burn injuries, especially severe ones, are proving to have devastating effects on the affected patients. Stem cells have been recently applied in the field to promote superior healing of the wounds. Not only have stem cells been shown to promote better and faster healing of the burn wounds, but also they have decreased the inflammation levels with less scar progression and fibrosis. This review aims to highlight the beneficial therapeutic effect of stem cells in burn wound healing and to discuss the involved pathways and signaling molecules. The review covers various types of burn wound healing like skin and corneal burns, along with the alternative recent therapies being studied in the field of burn wound healing. The current reflection of the attitudes of people regarding the use of stem cells in burn wound healing is also stated.

## 1. Introduction

The use of stem cell therapy is the yet to be discovered gold mine of science. A myriad of studies using stem cells are being done with promising results in various fields ranging from oncologic and hematologic diseases to organ transplants and wound healing. In the field of wound healing, the use of different types of stem cells has been reported for different types of wounds [1–3]. Burn wounds were of special interest due to the large number of cases of burns encountered nowadays, especially in the Middle Eastern Region and specifically in those areas with armed conflicts. Burn wounds have proven to be capable of having a devastating effect both functionally and cosmetically, necessitating the search for a better and more efficient cure. Being a very hot topic in the present field of research with constant studies and updates necessitated an updated review that encompasses the recent advances in stem cell therapy for burn wound healing in addition to relevant experimental studies. The literature was searched using the key words burn, stem cells, and wound healing. CINAHL, PubMed, EMBASE, and Medline were used as search engines to broaden the resources. The studies reported were not limited neither to humans nor by language and were mostly on animals unless otherwise specified. They are mostly reported in a chronological order of their publication dates, except when found relevant to group and mentioning some related studies consecutively.

Stem cells are undifferentiated pluripotential cells that are capable of producing other types of cells, including new stem cells identical to mother cells [4]. Stem cells can be of embryonal origin or adult origin, depending on the type of tissue they are derived from [4]. Embryonal stem cells are derived from either embryonal tissue or from germ cells in adults [4]. On the other hand, adult stem cells are derived from adult tissues of different organs, especially those with a high turnover rate such as intestines and bone marrow [4].

## 2. Wound Healing

Stem cells have been implicated in the healing of wounds in general. However, the methods of application of the stem cells in burn wound healing are diverse, including topical application, local injection, intravenous or systemic injection, and dermal or carrier application. Several studies have shown the efficacy of stem cells in promoting faster and superior wound healing. Alexaki et al. [5] successfully used adipose derived mesenchymal stem cells in wound healing in mice and compared their effect with dermal fibroblasts. The application of stem cells in wounds promoted more efficient reepithelialization by their proliferative effect on keratinocytes [5]. Moreover, this effect of stem cells was found to be mediated by keratinocyte growth factor-1 (KGF-1) and platelet derived growth factor-BB (PDGF-BB) [5]. Amniotic fluid derived stem cells have also been used in wound healing. Skardal et al. [6] tested the effect of amniotic fluid derived stem cells in wound healing in a mouse model. Wound closure, reepithelialization, and angiogenesis were more rapid in mice treated with the stem cells in comparison to those treated with fibrin collagen gel only [6]. Additionally, stem cells did not integrate permanently in the tissue, thus, suggesting that their effect is due to released factors and not by direct interaction [6]. Additionally, bone marrow derived mesenchymal stem cells have also been used in wound healing. Leonardi et al. [7] utilized bone marrow derived stem cells in artificial dermal substitutes to promote wound healing. These stem cells were shown to increase vascular density in the wounds along with the rate of reepithelialization [7]. A study by Zhang et al. [8] examined the effect of activin signaling on the homing of stem cells to wound sites. It was also found that JNK and ERK signaling pathways were involved in activin signaling and eventually the homing of stem cells [8].

## 3. Physiology of Burn Wound Healing

Concerning the physiology by which stem cells enhance the process of burn wound healing, several studies have been reported. Mansilla et al. [9] found evidence of cells in the bloodstream with identical phenotypes to mesenchymal bone marrow stem cells after acute large skin burns. Hence, it was concluded that these stem cells may have a role in promoting wound healing in burns. In a similar study, Fox et al. [10] reported increased levels of bone marrow derived endothelial progenitor cells in burn patients. These levels were proportional to the extent of the burn. The study also showed increased levels of angiogenic cytokines which may be involved in the signaling pathway for promoting the release of bone marrow derived stem cells. Focusing on the role of cytokines in burn wound healing, Payne et al. [11] studied the role of amnion derived cellular cytokine solution. In the study, Payne et al. used amnion derived multipotent progenitor cells to harvest cytokines and apply them in burn wound healing. Amnion derived cellular cytokine solution showed statistically significant improvement in the epithelialization of the burn wounds and the appearance of hair growth compared to controls [11]. In addition, the results demonstrated a faster epithelialization in burn wounds with increased frequency of application of the cytokines, further strengthening the role of stem cell derived cytokines in burn wound healing [11]. Furthermore, Foresta et al. [12] reported a positive linear correlation between endothelial progenitor cell blood levels and the total body surface area burnt. There was an increased level of endothelial progenitor cells in the bloodstream after escharectomy, posing a possible role of escharectomy in burn wound healing [12]. Additionally, stem cells could work by the release of bioactive peptides as proposed by Cabrera et al. [13] in their study where they showed that stem cells have an active role in burn wound healing by producing bioactive peptides, such as thymosin 4 and others.

More recent studies have also highlighted the role of stem cells in the process of wound healing in general and burn wound healing in specific. Koenen et al. [14] isolated acute wound fluids and chronic wound fluids and compared their effects on adipose tissue derived stem cell function in wounds. They came to the conclusion that acute wound fluids had a positive effect on the proliferation of adipose derived stem cells in wounds [14] while chronic wound fluids had a negative effect; the mentioned findings might explain the insufficient and slow healing process in chronic wounds due to a stem cell deficiency [14]. Furthermore, stem cells have been shown to decrease dermal fibrosis development in burn wound healing in mice [15]. Wu et al. performed a series of experiments which showed that bone marrow derived mesenchymal stem cells stimulate the formation of a basket weave organization of collagen in bleomycin treated skin, similar to normal skin [15]. Additionally, stem cell treatment of the skin decreased markers of myofibroblasts and downregulated type I collagen, leading to a decrease in the fibrosis that could have occurred to the skin [15]. Consequently, the role of stem cells in decreasing bleomycin induced fibrosis may be extrapolated to decrease fibrosis in burn wounds and improve their healing with less scar formation. Moreover, Lough et al. [16] performed a study in mice which showed a role for intestine derived human alpha defensin 5 in enhancing wound healing and decreasing its bacterial load. It induces leucine-rich repeat-containing G-protein-coupled receptors which are markers of adult epithelial stem cells both in skin and intestine [16]. Also implicated in the role of stem cells in burn wound healing is the role of SDF-1/CXCR4 signaling; Ding et al. [17] used interferon a2b in patients with burn wounds to suppress SDF-1/CXCR4 signaling. They found out that the decreased levels of signaling lead to better remodeling of hypertrophic scarring in the wounds [17]. Additional studies on the CXCR4 signaling pathway were done by Yang et al. [18] on irradiated mice. The mice having an overexpression of CXCR4, a receptor involved in the homing and migration of several stem cell types, showed an accelerated wound healing time [18]. Furthermore, Hu et al. [19] injected bone marrow derived mesenchymal stem cells into mice and studied the effect of blocking CXCR4 receptors. They found out that blocking the CXCL12/CXCR4 pathway, leading to activation of CXCR4, caused delayed wound closure in inflicted burn wounds. Moreover, CXCL12 levels were elevated in the burn wound one week after injury [19]. Hence, stem cells seem to be attracted to and attach to the burnt injury site by the CXCL12/CXCR4 pathway involving the CXCR4 receptors [18, 19]. The role of the ligand for the CXCR4 receptors, stromal cell derived factor-1 alpha (SDF-1a), has also been studied. Lü et al. [20] performed a study on the role of SDF-1a and its relation to the expression of miR-27b. It was found that SDF-1a expression was suppressed by direct binding of the miRNA to its 39UTR site [20]. As expected, miRNA expression was suppressed in wounds hence allowing better SDF-1a signaling and more homing of stem cells to the burn wounds [20]. In particular, miR-27b was found to be involved in the burn margins of wounds and in the mobilization of stem cells to the epidermis [20]. Chen et al. [21] performed experiments using porcine acellular dermal matrix on rats with inflicted 2nd degree burns. It stimulated collagen synthesis and stem cell proliferation and differentiation; porcine acellular matrix treated rats had a better and faster healing of the wounds.

Thus, in brief, the process of burn wound healing involves different types of growth factors, receptors, and cytokines. These factors are related to stem cell homing, differentiation, and proliferation. Additionally, when applied to burn wounds, they led to a better and faster healing process.

## 4. Stem Cells and Burn Wound Healing

The use of stem cells for burn wound healing, as reported in the literature, dates back to 2003 with Shumakov et al. [22]. Shumakov et al. were the first to use mesenchymal bone marrow derived stem cells (BMSC) in burn wound healing and compared them to embryonic fibroblasts [22]. The experiments were done on rats where mesenchymal bone marrow derived stem cells were applied to wounds showing decreased cell infiltration of the wound and an accelerated formation of new vessels and granulation tissue in comparison with embryonic fibroblasts and controls (burn wounds with no transplanted cells) [22]. Hence, this study marked a new era in the research of burn wound healing by being the first to test the use of stem cells in this complex process. Following this, a study by Chunmeng et al. [23] found that systemic transplantation of dermis derived multipotent cells promoted the healing of wounds in irradiated rats compared to controls with no transplantation, noting that topical transplantation of the cells had no superior effect. In 2004, Rasulov et al. [24] were the first to report using bone marrow mesenchymal stem cells in humans; a female patient with extensive skin burns (IIIB 30% of body surface area) had the stem cells applied onto the burn surface. The application of stem cells caused faster wound healing and active neoangiogenesis [24]. Another study done by Rasulov et al. on rats also showed the superiority of stem cells in burn wound healing [25]. In the rat study, the application of mesenchymal stem cells on burns reduced cell infiltration, improved neoangeogenesis, and reduced the formation of granulation tissue [25]. The aforementioned conditions created a better medium for wound healing in burns. In a similar effort, Liu et al. [26] performed experiments on pigs where they applied collagen scaffolds with seeded mesenchymal stem cells onto the surface of inflicted burns; the latter were found to induce better burn wound healing with less contraction and better vascularization and keratinization. Moreover, in human cutaneous radiation wounds, Lataillade et al. [27, 28] reported two cases where stem cells where used to aid in burn wound healing. Mesenchymal stem cells were applied, in addition to surgical excision, flaps, and grafts, to burn wounds of cutaneous radiation patients. In these patients, the application of the mesenchymal stem cells decreased the levels of inflammation and promoted a better healing [27, 28]. Further on the role of stem cells in irradiated skin were the studies conducted by Dong et al. [29, 30], where they additionally inserted a vector of human beta defensin 2 into the stem cells. The mentioned studies showed a positive role for stem cells transfected with beta defensin 2 in burn wound healing by exhibiting antibacterial properties in infected burn wounds [29, 30]. In a similar experiment, Ha et al. [31] transfected mesenchymal stem cells with vectors of hepatocyte growth factor. The experiment, done on rats, compared the wound healing of a partial thickness burn treated with stem cells alone or stem cells transfected with hepatocyte growth factor [31]. The group treated with the transfected stem cells showed a significantly larger range of reepidermalization starting the first week, along with a thicker epidermis and lower content of collagen I at 3 weeks after burn [31]. In the same year (2010), Agay et al. [32] performed experimental studies by inflicting pigs with cutaneous radiation and studying the role of stem cells in the healing of the wounds. Intradermal mesenchymal stem cell injections were given locally in the affected area. They led to the accumulation of lymphocytes in the wound with better vascularization compared to controls (pigs with no injections of mesenchymal stem cells) [32]. Later on, Riccobono et al. [33] studied, in another experiment, the role of adipose tissue derived stem cells in the treatment of cutaneous radiation. Autologous, allogeneic, and acellular (empty, control) vehicles of adipose derived stem cells were grafted onto the burn wound areas [33]. Autologous but not allogeneic adipose derived stem cells were found to promote superior burn wound healing with no necrosis and decreased pain [33].

Aside to direct stem cell application to burn wounds, Kinoshita et al. [34] inflicted cutaneous radiation wounds to pigs and used expanders with and without basic fibroblast growth factor to determine their effect on burn wound healing. The group with basic fibroblast growth factor and expander showed greater proliferation of the dermis and epidermis along with increased neoangiogenesis [34]. Thus, basic fibroblast growth factor, which is known to promote the proliferation of mesenchymal stem cells, improved burn wound healing [34, 35].

In 2010, Yan et al. [36] studied the efficacy of porcine bone marrow derived mesenchymal stem cells combined with skin derived keratinocytes, both infected with recombinant retrovirus expressing human (h) platelet derived growth factor-A, in the healing of irradiated skin. The cells were loaded onto a cultured cutaneous substitute and compared their effect on healing with a cell-free cultured cutaneous substitute [36]. The substitute with cells stimulated faster healing, epithelialization, angiogenesis, and better granulation of the burn wound [36]. In another experiment, Collawn et al. [37] inflicted laser burn wounds to organotypic raft cultures. The burn wounds were treated with dermal grafts with and without adipose derived stromal cells [37]. The adipose derived stromal cell-containing grafts showed complete healing of the epidermis after two days, whereas the cell-free grafts still had areas of injury; hence, those stem cells had a role in promoting faster healing of the burnt areas [37]. More on cutaneous radiation treatment came from Xia et al. [38] who transfected human vascular endothelial growth factor 165 and human beta defensin 3 into bone marrow derived mesenchymal stem cells and used the cells to treat irradiated skin. The stem cell treated area, in comparison with cell-free controls, showed shorter healing times with better granulation and collagen deposition [38]. Additionally, Xue et al. [39] examined the effect of human mesenchymal stem cells in mouse models. Mice with inflicted burn wounds were injected locally, in the burn area, with the stem cells (controls injected cell-free injections) [39]. Wound healing was significantly faster when stem cells were included in the injection with an increased and denser neoangiogenesis [39]. Stem cell injections also had a role in resuming activity and regaining body weight more rapidly [39]. Similarly, Mansilla et al. [40] used mesenchymal stem cells in burn wound healing in pigs through an acellular dermal matrix embedded with anti-CD44 antibodies to promote homing and attachment of the stem cells [40]. This study concluded that the use of these dermal matrices with stem cells not only promoted better healing of the burn wound, but also stimulated the formation of hair follicles and regeneration of muscles and ribs [40].

Concerning stem cells from human umbilical cords, Liu et al. [41] studied the effect of human umbilical cord derived mesenchymal stem cells in the healing of severe burns inflicted in rats. The stem cells were intravenously injected into the affected rats [41]. Liu et al. found that the injection of the stem cells into the rats accelerated the wound healing compared to controls, decreased the count of inflammatory cells, downregulated interleukins 1 and 6, and increased the levels of interleukin 10 and TSG-6 [41]. Moreover, stem cell injected rats had increased neovascularization and VEGF levels [41]. Not only do stem cells promote faster wound healing in burns, but also they prevent the progression of burn injuries as showed by Singer et al. [42]. The latter performed an experiment while inflicting thermal burns to rats, with several rectangular burns on each rat separated by unburned interspaces [42]. Some of the rats received tail vein injections of mesenchymal stem cells, while others received saline injections [42]. After 7 days, all of the unburned spaces in the controls were necrotic [42]. However, 20% of the unburned spaces in rats with stem cells injections did not necrose [42]. Consequently, stem cells were also shown to play a possible role in the prevention of progression of burn injuries. Furthermore, in a study by Xu et al. [43], applying autologous bone marrow derived mesenchymal stem cells to grafted burn wounds, they demonstrated decreased contraction of the grafts.

In 2014, Yang et al. [44] attempted to integrate mesenchymal stem cells with fibrin glue into the dressing of burn wounds. They inflicted scald wounds on the back of rats and applied dressing with fibrin glue and stem cells in one group, fibrin glue only in the second, and no intervention in the third [44]. One month later, the treatment group with fibrin glue and stem cells showed significantly faster healing than the other two; moreover, this group had more proliferation of sebaceous glands and the appearance of hair follicle-like structures which were not present in the other groups [44]. In another experiment, Lough et al. [45] isolated leucine-rich repeat-containing G-protein coupled receptor 6 (LGR6+) epithelial stem cells from the adnexal compartment of the skin of mice. They injected the harvested stem cells locally into inflicted burn wounds [45]. The wounds injected with stem cells showed a better healing along with increased vascular endothelial growth factor, platelet derived growth factor, and epidermal growth factor levels [45]. Stem cell injection also promoted the formation of nascent hair follicles and better neoangiogenesis in the wounds of the affected mice [45].

On the other hand, it is very pertinent to report another study in 2014 by Loder et al. [46] where they also tested the effect of adipose derived stem cells in the treatment of burns. The mice with inflicted burns that received stem cells injections showed no significant difference in comparison to controls (received saline injections) with respect to proliferation and vascularization [46]. Nevertheless, the role of stem cells in burn wound healing is a dynamic field and still under extensive research.

## 5. Stem Cells and Corneal Burn Wound Healing

Another area of particular interest in the field of burn wound healing is the chemical burns of the cornea. In the year 2000, Dua and Azuara-Blanco [47] used autologous limbal stem cells for ocular surface reconstruction of the contralateral eye. It resulted in the formation of a better corneal surface with significant improvement in the vision and symptoms of the patients [47]. Several other experiments and trials using limbal stem cells showed similar results in inducing improvement of corneal healing and decreased neovascularization in both human (adults and children) and animal subjects [48–53]. In 2007, Oh et al. [54] studied the therapeutic effects of mesenchymal stem cells on corneas with chemical burns. They reported that mesenchymal stem cell media and mesenchymal stem cell culture media (without the stem cells) reduced the inflammation and promoted neovascularization of the corneas [54]. They were also found to reduce the infiltration of CD4 cells, as well as IL-6, IL-10, and TGF-B1 levels. It is to be noted that the direct application of the stem cells provided superior results in the healing process in comparison with the stem cell culture media [54]. Another study by Ye et al. [55] utilized cyclophosphamide to suppress inflammatory reactions and the release of bone marrow stem cells into circulation. In this study, rabbits were inflicted with corneal alkali injuries. It was found that rabbits with an unsuppressed bone marrow had significantly greater reepithelialization of the corneas with clearer surfaces [55]. Thus, this study revealed the role of bone marrow cells in enhancing the healing of corneal chemical wounds. Furthermore, Sel et al. [56] inflicted alkali wounds on the corneal surfaces of mice and treated the corneas with bone marrow derived stem cells, CD117+ cells, or medium only as control. Reepithelialization of the wounds in the treatment groups was significantly faster than the control, with no difference in corneal transparency. Stem cells and CD117+ cells were absent from corneas after healing, thus suggesting that soluble factors may be responsible for the effect of the applied cells [56]. In a different study by Rama et al. [57], limbal stem cells were cultured on fibrin and used in corneal burns; not only did stem cells promote a better healing but also they had maintained a superior healing at a follow-up of 10 years later. Several other studies showed comparable results where mesenchymal derived or adipose derived stem cells promoted faster recovery of the corneal epithelium and decreased neovascularization, inflammation, and oxidative injury; moreover, stem cells stimulated the formation of clearer cornea media in some experiments [58–61]. Additionally, Basu et al. [62–64], in 2011 and 2012, reported a series of studies concerning the use of limbal stem cells in corneal burn wound healing. In the first study, Basu et al. [62] used limbal stem cells in corneal burn wound healing and followed them by penetrating keratoplasty procedures. Good results were observed but they were not compared to controls. However, in the second study, Basu et al. [63] observed that 66% of patients who failed primary procedures of corneal repair and who were subjected to a secondary limbal stem cell transplant on the affected cornea had successful improvement of the corneal surface with no neovascularization at a follow-up of two years. Later on, Sangwan et al. [64] used limbal biopsies from unaffected eyes and cultured them on amniotic membranes as substrates. Similar results to previous experiments were obtained with avascular epithelialization of the new corneal surfaces [64]. Furthermore and as demonstrated by Huang et al. [65], the use of allograft transplants of limbal stem cells in corneal burn wound healing also resulted in improved avascular corneal healing without the need for systemic immunosuppression. Pellegrini et al. [66] studied the biological factors that affected the stem cells' role in corneal burn wound healing; the accurate number of stem cells used expressing high levels of the p63 transcription factor was shown to have important influence.

## 6. Alternative Therapies in Burn Wound Healing

Stem cells do seem to have a very promising role in the treatment of burn wounds; however, other therapies are being developed to improve the treatment. For example, Klinger et al. [67] used fat injections in severe burn wounds as a trial to improve burn wound healing in humans. They did get results showing scar improvement and enhancement of tissue regeneration, but their study was limited to a small population [67]. In other studies, Auxenfans et al. [68] investigated the role of keratinocytes in improving wound healing in burns. They reported that keratinocytes induced a more rapid burn wound healing [68]. On the other hand, stromal vascular fraction has been also shown to play a possible role in enhancing burn wound healing [69]. Atalay et al. used isolated stromal vascular fraction in burn wound healing. It stimulated an increase in vascular endothelial growth factor and reduced the inflammation with an improved fibroblastic activity [69]. Additionally, Hussein et al. [70] studied the effect of Botox injections on burn wounds healing and found that Botox increased fibroblasts, TGF-B, and TNF-alpha levels and decreased inflammation, thus improving burn wound healing. Another recent study by Zhang et al. [71] showed a beneficial effect of heat shock protein 90 alpha on burn wound healing. It promoted faster healing and less inflammation. In addition, several other studies have examined the effects of different factors and substances such as curcumin, mast cell chymase, and phenytoin with hypericin on burn wound healing with promising results and better wound healing [72–74].

Stem cells are commonly derived either from bone marrow, umbilical cord, adipose tissue, or skin. Natesan et al. [75] have even used debrided skin from severe burns as a source of stem cells for wound healing and regeneration. Hence, the adipose tissue that is discarded from burn wound debridement may now be of use for better wound healing. In addition, Natesan et al. [76], in another study, used isolated stem cells from debrided skin with fibrin and collagen based scaffolds. The dermal equivalents, created in the study, decreased wound contraction leading to a better matrix deposition and epithelialization [76]. Along the same line, van der Veen et al. [77] isolated mesenchymal stem cells from excised burn wound eschar. These stem cells showed similar abilities to adipose derived stem cells in differentiating into osteocytes, chondroblasts, and adipocytes [77].

A relatively recent approach by Li et al. [78] studied the role of electric fields in the migration of stem cells. They proved that epithelial stem cells migrate to the cathode in an induced electric field, knowing that endogenous electric fields exist naturally in wounds [78]. The migration of the stem cells was found to be proportional to the strength of the electric field and its duration, with the involvement of epidermal growth factor receptor and mitogen activated protein kinase-PI3K [78]. Hence, in addition to the use of stem cells in burn wounds, electric fields can be applied to the wounds to better direct their migration [78].

## 7. Discussion

The role of stem cells in wound healing has been shown to be performed through several pathways, such as JNK and ERK59, and with the involvement of different factors and mediators, such as KGF-1 and PDGF-BB [5, 8]. Additionally, this role could also be carried out by the released factors and not only by direct integration of the stem cells into the wound scaffold or matrix [6].

Stem cells in burn wound healing have been found to follow the same mechanisms. The increased levels of stem cells in burn wounds suggested a possible enhancing role in aiding in the healing process [9, 10, 12, 13]. However, a lack of consistency of the outcome was documented. Different experiments may have used different amounts of purified stem cells, or stem cells at different stages of replication or differentiation in vitro, leading to what may seem different results. In brief, this review depicted the improved healing with stem cells qualitatively rather than quantitatively. To really demonstrate the value of different stem cells in the process of burn wound healing, more studies need to be done under optimal and well controlled conditions, aiming to measure a quantifiable improvement. Additionally, the excess use of stem cells may lead to unwanted results, such as increased fibrosis and thicker healed epithelium. Whether the effect is observed as a result of direct stem cell proliferation, or other induced substances and cells, needs to be studied in the future. Moreover, the role of cytokines released by stem cells along with bioactive peptides such as thymosin 4 has been documented to mediate the beneficial effect of the stem cell application in burn wound healing. Further data refer the superior healing probably not to the direct integration of the stem cells into the wound [11, 13]. Acute wound fluids were also shown to have a role in promoting faster healing of burn wounds, similarly reinforcing the role of mediators released by stem cells. Additionally, human alpha defensin 5 and the CXCL12/CXCR4 pathway with its signal SDF-1a were found to be inducers of stem cells in burn wounds [16, 18, 20].

In addition, stem cells have been shown to decrease cell infiltration, wound contraction, fibrosis, scar progression, and inflammation of burn wounds. Moreover, they have been found to promote faster burn wound healing and angiogenesis along with better granulation and the formation of hair follicles and sebaceous glands [15, 22–45]. The studies reviewed showed positive results in both animal experiments and human trials, both in partial and full thickness injury burns. In addition, different ways of stem cell application have been used ranging from using stem cell scaffolds to systemic and intradermal injection [23, 25, 32]. Furthermore, the sources of stem cells used are multiple. They are derived from bone marrow, dermis, adipose tissue, and umbilical cords, among others [22, 23, 33, 41]. Stem cells have proved to be efficient not only in skin burns but also in corneal chemical burns, thus increasing the multiplicity of their use [54–61].

Patients with burn wounds, especially those severely injured, tend to have lower quality of life [79]. The injury they suffer is not only physical but also psychological, affecting their jobs and relations with other people, especially their families [80, 81]. With the advance of burn wound treatment with time, patients' self-esteem and quality of life have been improving [82, 83]. The hope is that the use of stem cells will open up a new arena of possibilities to improve the wound healing in burn patients, allowing patients to have faster healing, better scars, and a higher quality of life.

## 8. Reflections on the Role of Stem Cells in Burn Wound Healing

Stem cells have attracted many controversial public opinions over time. Many people argue that embryonic stem cell harvesting would be done by killing embryos which would be unethical [84]. Others would argue that even if embryos are used for stem cell research, it is not wrong. However, the path that this may lead to would be wrong such as embryo “production” for research purposes [84]. The public view towards the therapeutic use of stem cells has become more tolerant over time [85]. The role of educating people about the colossal potential for the use of stem cell has thus proven beneficial. People are now more educated about the different sources of stem cells and have become supportive of their use [85]. Regarding the acceptance of stem cells as an efficient therapy for burn wound healing in specific, a study done by Clover et al. [86] showed a very positive opinion. The biggest majority of people were willing to accept autologous stem cells, though a big percentage was also welcoming the idea of using allogeneic stem cells. These percentages did not differ between the use of stem cells for burn wounds or for the treatment of other diseases such as diabetes or Parkinson's [86].

## 9. Conclusion

In brief, the use of stem cells in burn wound healing appears to be very promising. While most studies were performed on animals, the application to humans is yet at its start. Hence, what is needed is more studies. Additionally, the signaling pathways followed by stem cells involved in the burn wound healing along with their factors and signals constitute a very dynamic and promising research field.
